# Risk factors for patients hospitalized with recurrent colon diverticular bleeding: a single center experience

**DOI:** 10.3389/fmed.2023.1195051

**Published:** 2023-11-07

**Authors:** Hye-Su You, Dong Hyun Kim, Seo-Yeon Cho, Seon-Young Park, Chang Hwan Park, Hyun-Soo Kim, Sung Kyu Choi

**Affiliations:** Division of Gastroenterology and Hepatology, Department of Internal Medicine, Chonnam National University Medical School, Gwangju, Republic of Korea

**Keywords:** colon, comorbidity, diverticular diseases, diverticulum, recurrence

## Abstract

**Background/aims:**

Colonic diverticular bleeding (CDB) is a common cause of acute lower gastrointestinal bleeding. Patients with CDB are at increased risk for recurrence. Here, we aimed to evaluate the clinical course of patients with CDB and identify risk factors for recurrent CDB (rCDB).

**Methods:**

We included patients who were hospitalized at a single tertiary center for management of CDB between January 2005 and March 2020. A Cox proportional hazards regression analysis was performed to evaluate the risk factors of patients with rCDB as follows: model 1 adjusted by age, Charlson comorbidity index (CCI), and presence of bilateral colon diverticula; model 2 adjusted by age, CCI, and presence of left side colon diverticula; model 3 adjusted by age, CCI, and presence of sigmoid colon diverticula.

**Results:**

Among 219 patients (mean age, 68.0 years; 55 females), 56 and 163 had definite and presumptive CDB, respectively. During the median period of 506 days, 62 patients (28.3%) experienced rCDB. CCI score ≥ 4 was independently associated with rCDB in models 1, 2 and 3 (all *p* < 0.05). Age ≥ 75 years was independently associated with rCDB in models 1 and 2 (both *p* < 0.05). The presence of bilateral colon and sigmoid colon diverticula were independently associated with rCDB in models 1 and 3, respectively (both *p* < 0.05).

**Conclusion:**

rCDB frequently occurred at any time in patients with previous CDB. High CCI scores and distribution of colon diverticula were associated with rCDB. Clinicians should consider a possible rCDB for a patient considering age, comorbidity, and distribution of colon diverticula.

## 1. Introduction

Despite the decreasing incidence of upper gastrointestinal bleeding, the incidence of lower gastrointestinal bleeding (LGIB) is gradually increasing (1). Colonic diverticular bleeding (CDB) is a common cause of LGIB (2), and the risk factors include old age, male sex, obesity, non-steroidal anti-inflammatory drug (NSAID) or antithrombic agent (AT) use, and underlying comorbidities, including cardiovascular diseases, hypertension, or diabetes (3). Recurrent CDB (rCDB) occurs within 1 year at rates of approximately 4–35% (4–7). Despite several efforts, including avoiding or decreasing the number of probable triggers, rCDB may occur at various times and in clinical situations, leading to increased morbidities, rehospitalizations, and medical costs. The risk factors associated with rCDB seem to be similar but inconclusive, as previous studies included a small number of patients and a lack of long-term follow-up data, and related clinical situations at each period may differ. Therefore, studies with a larger number of completed long-term follow-up patients considering the overall clinical factors, including age, comorbidities, and prescribed medications, for patients with rCDB are needed. Therefore, we aimed to evaluate the clinical course of patients with CDB and identify risk factors for rCDB.

## 2. Methods

### 2.1. Study population

This study was conducted in accordance with the ethical guidelines of the Declaration of Helsinki and was approved by the institutional review board of Chonnam National University Hospital (IRB No.: CNUH-2020-115).

From January 2005 to March 2020, a total of 3,539 consecutive patients with gastrointestinal bleeding were admitted to our center, of which 1,572 had obscure GIB or LGIB. We excluded 1,351 patients with bleeding of small bowel origin, colon cancer, ischemic colitis, inflammatory bowel disease, hemorrhoids, angiodysplasia, colonic ulcers, or other conditions based on a medical chart review. We also excluded two patients who died on the first admission. Finally, 219 patients with CDB were included in this study (Supplementary Figure S1).

We retrospectively reviewed electronic medical records and extracted information on demographic factors and clinical characteristics, including comorbidities, medication history or laboratory findings, diagnostic or therapeutic procedural outcomes, and the presence and relevance of rCDB with mortality.

### 2.2. Assessment of comorbidity and Charlson comorbidity index score

The components of the Charlson comorbidity index (CCI) include the following: acquired immunodeficiency syndrome status, cerebrovascular accident, chronic obstructive pulmonary disease, congestive cardiac failure, connective tissue disease, dementia, diabetes, hemiplegia, leukemia, liver disease, lymphoma, peptic ulcer disease, peripheral vascular disease, previous myocardial infarction, renal disease, and solid tumor with or without metastasis. We calculated the CCI score by adding the weights of all comorbid parameters.

### 2.3. Definition of CDB

All patients underwent colonoscopy and abdominal computed tomography (CT) to exclude other diseases, such as colonic inflammatory bowel disease or cancer. Definite CDB was diagnosed using colonoscopic findings of stigmata of recent diverticular hemorrhage (active bleeding, visible vessel, or adherent clot). Presumptive CDB was diagnosed using endoscopic features of a diverticulum with fresh blood clots in the colon or abdominal CT findings of a colonic extravasation, and colonoscopic findings of a diverticulum without other bleeding foci. Additional examinations, including esophagogastroduodenoscopy, abdominal CT, and capsule endoscopy, cannot confirm other bleeding foci (4, 6).

### 2.4. Location of diverticula

The anatomical distribution of diverticula was divided into two groups for comparison in three ways: the presence or absence of right-sided colonic diverticula in the cecum, ascending colon, or transverse colon; the presence or absence of left-sided diverticula in the descending or sigmoid colon; bilateral or unilateral colonic diverticulosis.

### 2.5. Treatment method

Endoscopic hemostasis, endoscopic clipping (EC), epinephrine injection therapy, and argon plasma coagulation were used. Trans arterial embolization was also performed when extravasation was prominent on CT. If the bleeding was not controlled by endoscopic treatment or embolization, surgical treatment was performed.

### 2.6. Adjustment of antithrombotic agents

We classified antithrombotic agents (ATs) into antiplatelet agents and anticoagulants. Antiplatelet agents included aspirin, thienopyridine, and cilostazol. Anticoagulants included low-molecular-weight heparin (LMWH; enoxaparin and dalteparin), warfarin, and direct oral anticoagulants (DOAC; apixaban, edoxaban, and rivaroxaban). Decisions to use ATs during hospitalization and after discharge were made by the attending physician based on a medical judgment of necessity. We defined ‘adjustment of ATs’ as the discontinuation of any AT during admission and after discharge.

### 2.7. Confirmation of death outside the hospital

The patient’s date of death and related disease codes were confirmed by request from the Korea National Statistical Agency. Disease codes were classified by the Korean Standard Classification of Disease and Cause of Death (KCD7) (8, 9).

### 2.8. Outcomes

The primary outcomes were the risk factors of patients with rCDB. rCDB was defined as a significant amount of fresh bloody or wine-colored stool with hemodynamic instability, a need for transfusion, identification of stigmata of recent hemorrhage on repeated colonoscopy, or identification of extravasation in the colonic diverticula on repeated abdomen CT after discharge. The secondary outcomes were the rebleeding rates and the cause of death in patients with CDB.

### 2.9. Statistical analysis

Continuous and categorical data are expressed as means ± standard deviations or medians (ranges) and absolute or relative frequencies, respectively. Continuous variables were analyzed using a Student’s *t*-test. Categorical data were examined using the Fisher exact test or χ^2^ test. Rebleeding-free days were calculated from the date of the first bleeding to the date of recurrence or death using the Kaplan–Meier method. Patients without evidence of recurrence were classified as alive and event-free at the date of the last follow-up. We performed a univariate analysis using the log-rank test. Variables with a value of *p* < 0.05 were analyzed by multivariate analysis using a Cox proportional hazard model to evaluate the risk factors associated with rCDB. In all statistical tests, a two-sided value of *p* of <0.05 was considered statistically significant. The Kaplan–Meier analysis was performed using GraphPad Prism (version 5.0; GraphPad Software Inc., San Diego, CA), and all other analyses were performed using SPSS (version 25.0; SPSS Inc., Chicago, IL).

## 3. Results

### 3.1. Baseline demographic and clinical characteristics

A total of 219 patients (mean age, 68.0 ± 12.4 years; 55 females) were enrolled in the study. The baseline characteristics of the 219 patients are described in Supplementary Table S1. The most common comorbidities were hypertension (62.4%) and diabetes (33.3%). ATs and NSAIDs were administered to 109 (49.8%) patients and 22 (10.0%) patients, respectively. There were 86 (39.3%) patients with a CCI score of 0 and 133 (60.7%) with a CCI score ≥ 1. Comorbidities contributing to CCI are shown in Supplementary Table S2.

Definite and presumptive CDB were diagnosed in 56 (25.6%) and 163 (74.4%) patients, respectively. Among the 219 patients, right-sided colon diverticula were observed in 189 (86.3%), left-sided colon diverticula were observed in 87 (39.7%), and bilateral colon diverticula were observed in 57 (26.0%).

Fifty-three (24.2%) patients experienced shock at admission, and 164 (74.9%) underwent urgent colonoscopy within 24 h of admission. Cecal intubation was successfully performed in 208 (95.0%) patients. While bleeding stopped spontaneously in 152 (69.4%) patients, urgent endoscopic, radiologic, and surgical interventions were performed in 55 (25.1%), 10 (4.6%), and 2 (0.9%) patients, respectively. The median duration of hospitalization was 6 days.

### 3.2. Risk factors of rCDB

**Figure 1:**
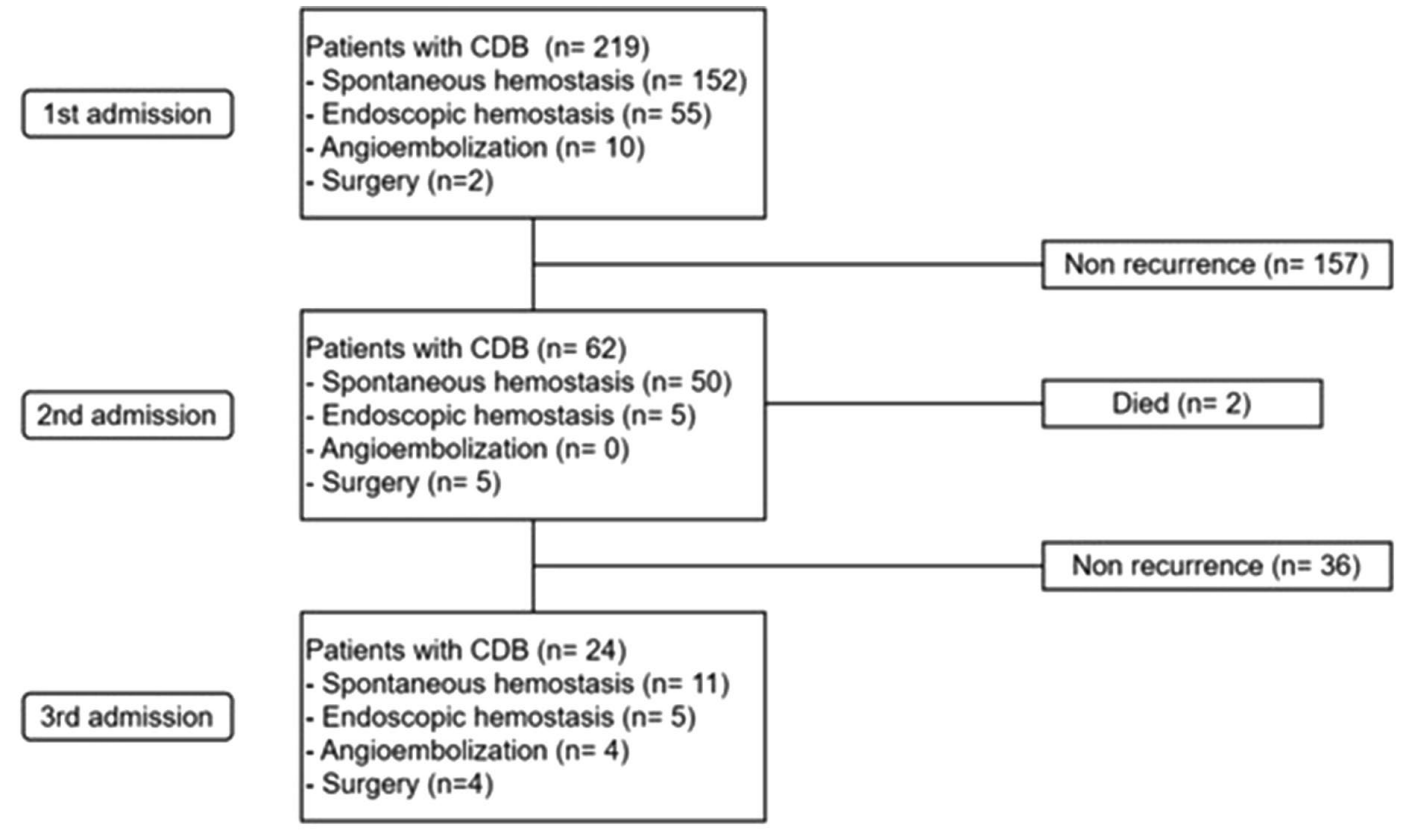
Long-term outcomes of re-admission and therapeutic modalities in study population. CDB, colonic diverticular bleeding.

During the median follow-up of 60 months (range, 0.1–182.4), rebleeding occurred in 28.3% (62/219) of patients, and third rebleeding occurred in 11.0% (24/219) of patients (Figure 1). The cumulative incidence rates of rCDB at 1, 6, 12, and 24 months were 5.0% (11/219), 9.1% (20/219), 12.8% (28/219), and 17.4% (38/219), respectively.

**Figure 2:**
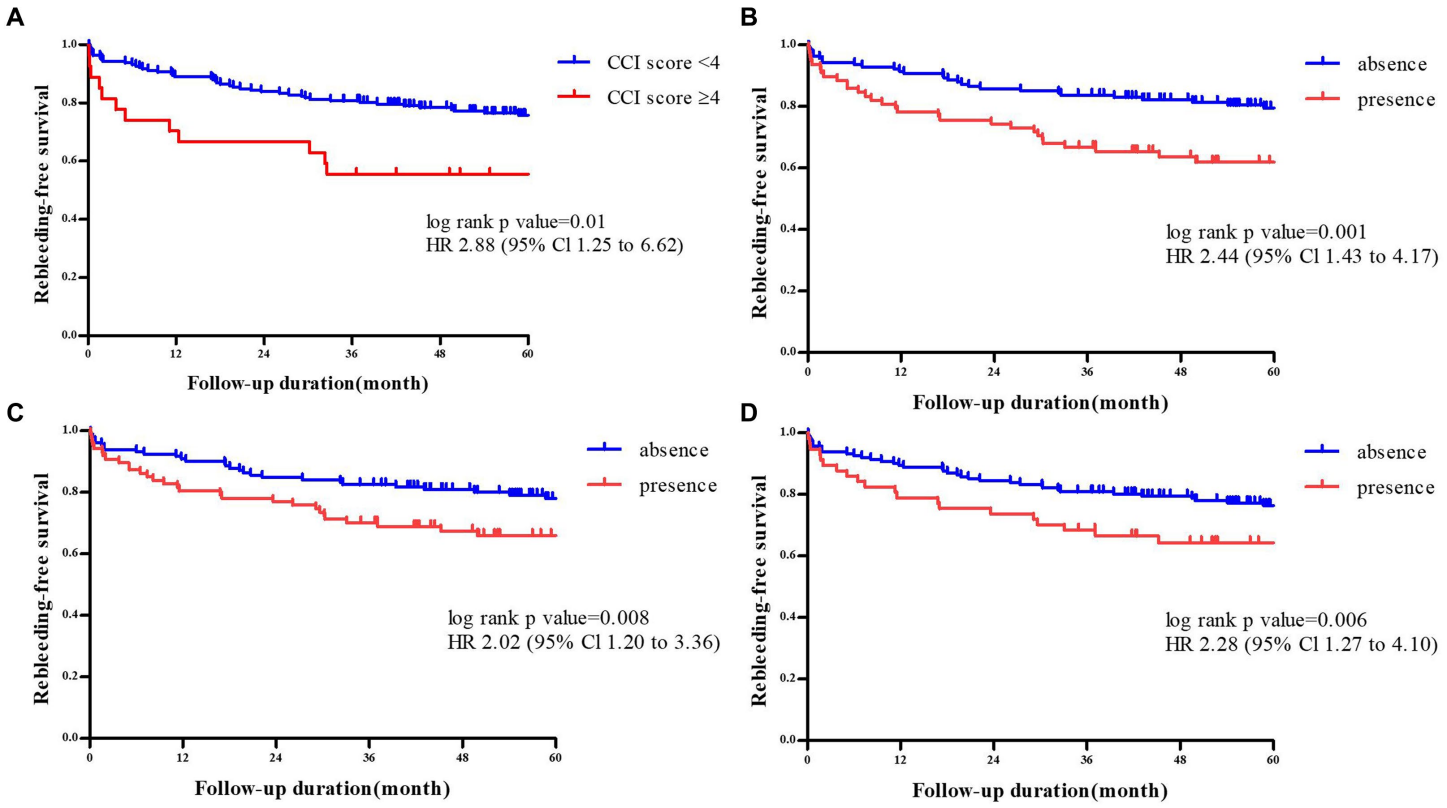
Comparison of rebleeding -free survival rate according to the Charlson comorbidity index scores of≥ 4 or <4 **(A)**, the presence of sigmoid colon diverticuli **(B)**, the presenceof left colonic diverticuli **(C)**, and bilatral diverticuli **(D)**.

Table 1 shows the univariate analysis for risk factors of rCDB, in which age ≥ 75 years (*p* = 0.008), CCI score ≥ 4 (*p* = 0.015), and location of diverticula [the presence of bilateral colon diverticula (*p* = 0.007), left colon diverticula (*p* = 0.010), and sigmoid colon diverticula (*p* = 0.001)] were associated with rCDB. There were no significant differences between the two groups in terms of sex, underlying comorbidities, laboratory findings, use of ATs or NSAIDs, and hemostatic treatments (all *p* > 0.05) (Table 1). The Kaplan–Meier analysis showed that rCDB occurred more in patients with CCI scores ≥4 and in those with a presence of sigmoid colon, left colonic, and bilateral diverticulosis (All *p* ≤ 0.01) (Figure 2).

**Table 1:** Univariate analysis of risk factors for recurrent colonic diverticular bleeding.

Variables	Patients with rCDB (*n* = 62)	cHR	95% CI	Value of *p*
Age				0.008
Age < 75 (*n* = 139)	31 (22.3)	1		
Age ≥ 75 (*n* = 80)	31 (38.8)	1.96	1.19–3.22	
Females, *n* (%)	17 (27.4)	1.24	0.71–2.17	0.450
Body mass index, kg/m^2^, mean ± SD	24.7 ± 3.6	1.03	0.96–1.11	0.450
Diagnosis, *n* (%)				
Definite (*n* = 56)	17 (27.4)	1.06	0.61–1.85	0.838
Presumptive (*n* = 163)	45 (72.6)	1		
Location of diverticula, *n* (%)				
Presence of bilateral colon diverticula				0.007
No (*n* = 162)	38 (23.5)	1		
Yes (*n* = 57)	24 (42.1)	2.03	1.21–3.38	
Presence of right colon diverticula				0.774
No (*n* = 30)	9 (30.0)	1		
Yes (*n* = 189)	53 (28.0)	0.90	0.44–1.83	
Presence of left colon diverticula				0.010
No (*n* = 132)	29 (22.0)	1		
Yes (*n* = 87)	33 (37.9)	1.94	1.18–3.19	
Presence of descending colon diverticula				0.122
No (*n* = 175)	45 (25.7)	1		
Yes (*n* = 44)	17 (38.6)	1.55	0.89–2.72	
Presence of sigmoid colon diverticula				0.001
No (*n* = 141)	30 (21.3)	1		
Yes (*n* = 78)	32 (41.0)	2.25	1.37–3.71	
Charlson comorbidity index. *n* (%)				0.015
≤3 (*n* = 192)	50 (26.0)	1		
≥4 (*n* = 27)	12 (44.4)	2.18	1.16–4.11	
Laboratory findings, median (IQR)				
Hemoglobin (mg/dL)	9.9 (8.7–11.8)	0.95	0.86–1.05	0.318
Platelet count (mm3)	194 (163–243.5)	1.00	1.00–1.01	0.093
PT (INR)	1.1 (1.0–1.1)	1.18	0.90–1.55	0.240
eGFR	86 (67.4–99.3)	0.99	0.98–1.00	0.071
Use of anti-thrombotic agents				0.481
No (*n* = 110)	29 (26.4)	1		
Yes (*n* = 109)	33 (30.3)	1.20	0.73–1.97	
Adjustment of anti-thrombotic agents (among 109 patients taking anti-thrombotic agents)				0.100
No (*n* = 85)	29 (34.1)	1		
Yes (*n* = 24)	4 (16.7)	0.42	0.15–1.18	
Use of NSAIDs				0.347
No (*n* = 197)	58 (29.4)	1		
Yes (*n* = 22)	4 (18.2)	0.62	0.22–1.70	
Hemostatic treatment				0.999
No (*n* = 157)	44 (28.0)	1		
Yes (*n* = 62)	18 (29.0)	1.00	0.58–1.73	

rCDB, recurrent colonic diverticular bleeding; cHR, crude hazard ratio; CI, confidence interval; SD, standard deviation.

We performed a multivariate analysis including variables with a value of *p* <0.05 (age ≥ 75 years, CCI score ≥ 4), considering the location of colon diverticula (the presence of bilateral colon, left side colon, and sigmoid colon diverticula): model 1 adjusted by age, CCI, and the presence of bilateral colon diverticula; model 2 adjusted by age, CCI, and the presence of left side colon diverticula; model 3 adjusted by age, CCI, and the presence of sigmoid colon diverticula.

Model 1 showed that an age ≥ 75 years, a CCI score ≥ 4, and the presence of bilateral colon diverticula were all independent risk factors for rCDB (adjusted hazard ratio [aHR] 1.70, 95% confidence interval [CI] 1.02–2.83, *p* = 0.04 for age ≥ 75 years; aHR 2.24, 95% CI 1.19–4.23, *p* = 0.01 for a CCI score ≥ 4; aHR 2.24, 95% CI 1.19–4.23, *p* = 0.01 for the presence of bilateral colon diverticula). Model 2 showed that age ≥ 75 years and a CCI score ≥ 4 were both independent risk factors for rCDB (aHR 1.92, 95% CI 1.17–3.16, *p* = 0.01 for age ≥ 75 years; aHR 2.11, 95% CI 1.12–3.98, *p* = 0.02 for a CCI score ≥ 4). Model 3 showed that a CCI score ≥ 4 and the presence of sigmoid colon diverticula were both independent risk factors for rCDB (aHR 2.48, 95% CI 1.31–4.68, *p* < 0.01 for a CCI score ≥ 4; aHR 2.41, 95% CI 1.46–3.98, *p* < 0.01 for the presence of sigmoid colon diverticula) (Table 2).

**Table 2:** Cox proportional hazards models of risk factors for recurrent colonic diverticular bleeding.

	aHR	95% CI	Value of *p*
Model 1			
Age ≥ 75	1.695	1.016–2.828	0.043
CCI score ≥ 4	2.243	1.188–4.233	0.013
Presence of bilateral colon diverticula	1.885	1.114–3.190	0.018
Model 2			
Age ≥ 75	1.919	1.165–3.160	0.010
CCI score ≥ 4	2.114	1.123–3.979	0.020
Presence of left colon diverticula			
Model 3			
Age ≥ 75			
CCI score ≥ 4	2.476	1.309–4.682	0.005
Presence of sigmoid colon diverticula	2.407	1.456–3.980	0.001

aHR, adjusted hazard ratio; CCI, Charlson comorbidity index; CI, confidence interval.

### 3.3. Adjustment of ATs in patients taking ATs

Among 109 (49.8%) patients that were taking ATs, 24 (22.0%) discontinued AT agents after discharge upon medical judgment by an attending physician. A Kaplan–Meier analysis showed that rCDB occurred less in patients with adjustment of ATs than in patients who continued using ATs, but this was statistically insignificant (HR 1.98, 95% CI 0.90–4.37 *p* = 0.08) (Supplementary Figure S2).

### 3.4. Causes of death

Two patients died at the first admission because of diverticular bleeding and acute myocardial infarction, respectively. During the follow-up after discharge, 31 out of the remaining 219 patients died, of which 11 (21.6%) had rCDB and 20 (11.8%) did not have rCDB (*p* = 0.08). One patient with rCDB had a CDB-related death. The most common cause of death during the follow-up was pneumonia (*n* = 5, 16.1%) (Supplementary Table S3).

## 4. Discussion

The incidence of CDB is increasing because of the increase in colonic diverticulosis, an aging population, and AT use (3). Approximately 70–90% of diverticular bleeding events spontaneously stop, complicating the definite diagnosis of diverticular bleeding (10–12). In our study, only 25% of patients were diagnosed with definite CDB, which was similar to proportions in previous studies (19–42%) (6, 13, 14), and CDB spontaneously stopped in 69.4% of patients. Despite this, about 25% of the patients with CDB experienced hypovolemic shock at admission, which was also similar to a previous study (25.6%) (7). Therefore, any changes in symptoms or vital signs should be closely monitored, and adequate resuscitation should be provided for all patients with CDB. This study presented the clinical course of 219 patients with CDB over 15 years in a tertiary referral center. The recurrence rate was as high as 28.3%.

The CCI was developed to determine the association between patients’ various underlying diseases and one-year mortality (15). The CCI is helpful in classifying patients by weighing their comorbidities and has been validated in various disease subgroups, such as cardiac, renal, stroke-related, and liver diseases (15–19). Recently, the CCI has been used to predict the prognosis in various patient groups (16–20), with high CCI scores becoming a risk factor for severe CDB (21). In this study, we used the CCI score to reflect the overall status of comorbidities. The incidence of rCDB was about two times higher in patients with CCI scores ≥4 after adjusting factors found to be significant in a univariate analysis, such as age and location of diverticula. Therefore, clinicians should consider the occurrence of rCDB and make active efforts, such as controlling underlying diseases and adjusting the related medication in patients with diverticular bleeding, especially in patients with a high CCI score.

While left-sided colonic diverticulosis is more common in Western countries, right-sided or bilateral colon diverticula are more common in Asian countries (13, 22–25), which was similar to our study. Over 95% of patients in our study underwent colonoscopy with a full examination, which allowed the precise detection of the anatomical distribution of diverticulum. Interestingly, our study identified that the location of the colon diverticula was associated with the occurrence of rCDB. Patients with bilateral colon diverticula had a higher occurrence of rCDB, which was similar to previous studies (24, 26, 27). We also demonstrated that patients with sigmoid colon diverticula had a higher occurrence of rCDB. Left-sided colonic diverticulosis is considered acquired rather than congenital and increases with age (28). However, even after adjustment for significant factors, such as CCI and age, sigmoid colon diverticulosis was an independent risk factor for rCDB. Colonoscopy, especially performed in an emergency situation, is not perfect due to inadequate bowel preparation and remaining bloody clots attached to the multiple diverticular sacs and colonic mucosa. This limited surface visualization may lead to blind spots, especially in the flexure, behind folds, and angulated area of the colon. Compared to other locations of the colon, it can be difficult to find the bleeding focus from sigmoid colon diverticula using a colonoscope due to anatomical characteristics, such as the many folds and angulation of the sigmoid colon. In addition, the sigmoid colon length can vary depending on the endoscopist’s colonoscopic manipulation. This length ranges from 40 to 70 cm when stretched during scope insertion and is shortened to only 30–35 cm when the scope is straightened fully (29). For these reasons, endoscopists may miss the hidden bleeding focus in the sigmoid colon, leading to rCDB. Further studies are needed to elucidate the relationship between the location of colon diverticula and rCDB.

Endoscopic hemostasis can achieve a high rate of active bleeding control (30, 31) and is typically considered the first-line treatment for CDB management (32, 33). In this study, 75% of patients had undergone urgent colonoscopy examinations within 24 h. Although aggressive colonoscopic evaluations were performed as the first evaluation, an endoscopic intervention was performed in only a third of the patients. A literature review reported an endoscopic hemostasis rate ranging from 20.8 to 32.8% in Western countries (34, 35) and 16.8 to 34.1% in Eastern countries (36, 37), which were similar to the rates in our study. Angiographic treatment and surgical resection are other modalities used if endoscopic hemostasis fails (38, 39). In our study, radiological interventions were performed in 11 (5.0%) patients and surgical intervention in two (0.9%). While the most of diverticular bleeding episodes resolve spontaneously with conservative treatment, surgical treatment becomes necessary under specific circumstances, including persistent bleeding, recurrent bleeding episodes, and even the development of hypovolemic shock (40, 41). Urgent surgery is required in approximately 10 to 25% of patients who experience hemodynamic instability (42). Studies have reported morbidity and mortality rates of 17 and 8.3%, respectively, among patients with acute CDB who underwent urgent surgery (43). Recent research has underscored that the presence of comorbid diseases is an independent risk factor for requiring urgent colectomy for CDB, with both mortality and morbidity rates as high as 20% (40). Consequently, patients with more comorbid diseases may face increased risks when undergoing urgent colectomy.

Although NSAIDs, steroids, ATs, obesity, hypertension, and chronic kidney disease are risk factors for rCDB, each study showed inconsistent results (4–6, 44). In this study, medications, including ATs, NSAIDs, and steroids, did not increase the risk of rCDB. However, rCDB occurred less in patients with an adjustment of ATs than in patients with a continuation of ATs. Thus, the modification of ATs should be considered to prevent rebleeding. However, it is possible that the discontinuation of ATs could result in fatal thromboembolic events, particularly in elderly patients. Therefore, we believe that the discontinuation of ATs should be carefully considered along with their risks and benefits.

In this study, CDB-related death occurred in two patients, and the overall mortality rate was twice as high in patients with rCDB compared to those without rCDB. Although CDB itself was not the immediate cause of death, increased hospitalization and morbidity due to CDB may have affected the increase in mortality.

Our study had two limitations. First, this was a retrospective cohort study conducted at a single referral center. However, a larger number of cases were evaluated in this study than in previous studies. Second, this study did not reflect the effects of variable endoscopic hemostatic methods, such as endoscopic band ligation, endoscopic detachable snare ligation, use of topical hemostatic agents, and over-the-scope clip (36, 45–47). We typically performed endoscopic hemostasis using endoscopic clipping or endoscopic injection therapy. Recent studies showed that endoscopic band ligation was more helpful in decreasing early rCDB (13, 39).

## 5. Conclusion

rCDB frequently occurred at any time in patients with previous CDB. High CCI scores and distribution of colon diverticula were associated with rCDB. Clinicians should consider a possible rCDB for a patient considering age, comorbidity, and distribution of colon diverticula.

## Data availability statement

The raw data supporting the conclusions of this article will be made available by the authors, without undue reservation.

## Ethics statement

The studies involving humans were approved by the institutional review board of Chonnam National University Hospital (IRB No.: CNUH-2020-115). The studies were conducted in accordance with the local legislation and institutional requirements. Written informed consent for participation was not required from the participants or the participants’ legal guardians/next of kin in accordance with the national legislation and institutional requirements.

## Author contributions

S-YP and H-SK developed the concept of the study, analyzed the collected data, wrote the manuscript, revised it critically for intellectual content and supervised the study. H-SY, S-YC, and DK collected clinical data, analyzed th electronic medical records, and wrote the manuscript. CP and SC collected clinical data. All authors have read and approved the final manuscript.
