# Supplementary material for: Risk factors for patients hospitalized with recurrent colon diverticular bleeding: a single center experience
Source: Front Med (Lausanne). 2023 Nov 7;10:1195051. doi: 10.3389/fmed.2023.1195051 (PMC10661954; doi:10.3389/fmed.2023.1195051)
Supplement: Supplementary file 1 [file Data_Sheet_1.docx]

***Supplementary Material***

**Risk Factors for Patients Hospitalized with Recurrent Colon Diverticular Bleeding: A Single Center Experience**

**Hye-Su You, Dong Hyun Kim, Seo-Yeon Cho, Jung Yong Wook, Seon-Young Park, Chang Hwan Park, Hyun-Soo Kim and Sung Kyu Choi**

*** Correspondence:** Seon-Young Park: drpsy@naver.com


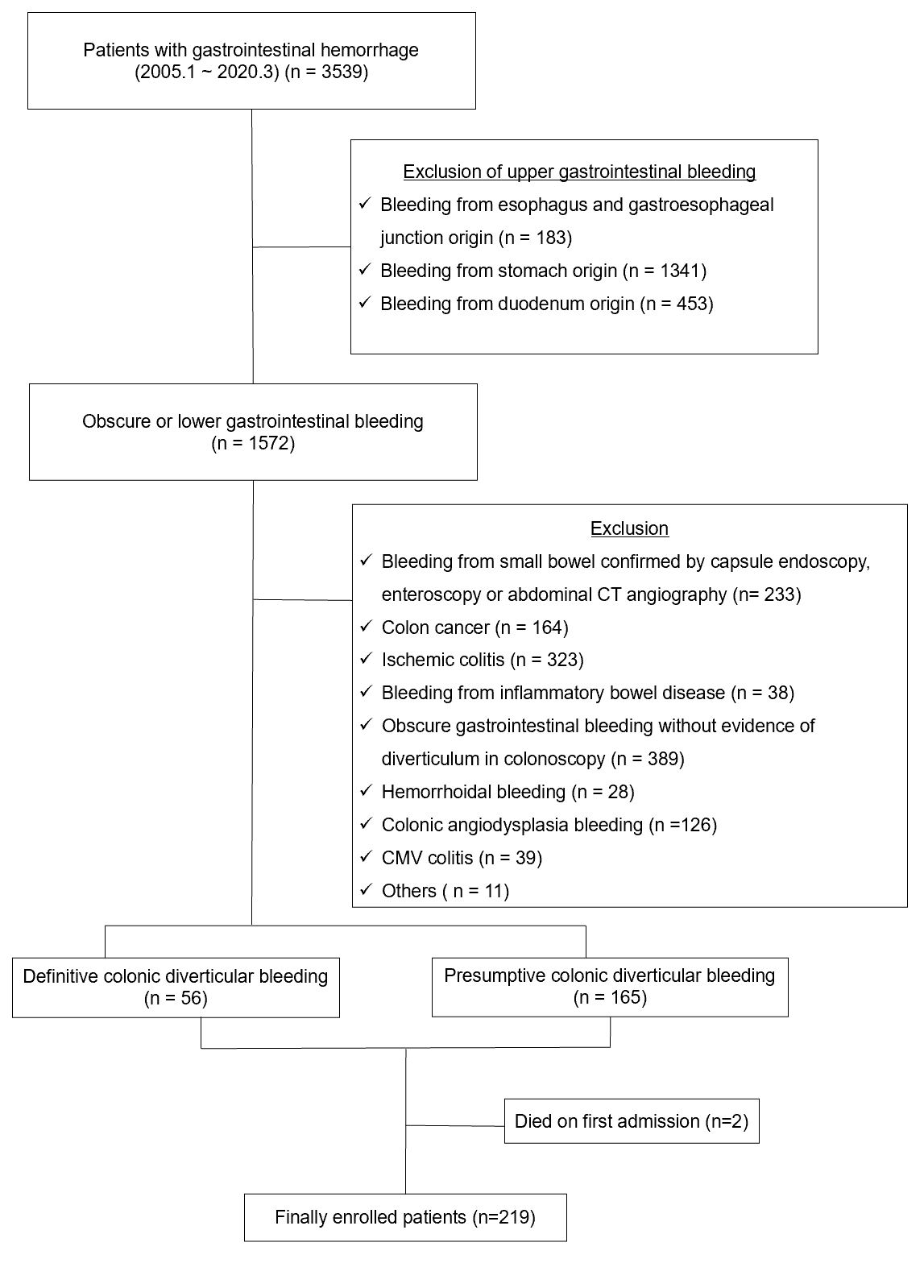


**Supplementary Figure 1.** Patient enrollment flowchart.

CMV, cytomegalovirus; CT, computed tomography.

**Supplementary Figure 2.** Comparison of rebleeding-free survival rate according to the adjustment of ATs in the AT taking group using the Kaplan-Meier graph.

AT, anti-thrombotic agent; CI, confidence interval; HR, hazard ratio

**Supplementary Table 1.** Baseline characteristics of patients with colonic diverticular bleeding

| Variables | Patients with rCDB (*n* = 62) | Patients without rCDB (*n* = 157) | *p*-value |
| --- | --- | --- | --- |
| Age, n (%) |  |  |  |
| Age < 75 (*n* = 139) | 31 (22.3) | 108 (77.7) | 0.009 |
| Age ≥ 75 (*n* = 80) | 31 (38.8) | 49 (61.3) |  |
| Females, *n* (%) | 17 (27.4) | 38 (24.2) | 0.62 |
| Body mass index, kg/m^2^, mean ± SD | 24.7 ± 3.6 | 24.3 ± 3.4 | 0.52 |
| Diagnosis, *n* (%) |  |  | 0.69 |
| Definite (*n* = 56) | 17 (27.4) | 39 (24.8) |  |
| Presumptive (*n* = 163) | 45 (72.6) | 118 (75.2) |  |
| Location of diverticula, *n* (%) |  |  |  |
| Presence of bilateral colon diverticula |  |  | 0.007 |
| Yes (*n* = 57) | 24 (42.1) | 33 (57.9) |  |
| No (*n* = 162) | 38 (23.5) | 124 (76.5) |  |
| Presence of right colon diverticula |  |  | 0.825 |
| Yes (*n* = 189) | 53 (28.0) | 136 (72.0) |  |
| No (*n* = 30) | 9 (30.0) | 21 (70.0) |  |
| Presence of left colon diverticula |  |  | 0.010 |
| Yes (*n* = 87) | 33 (37.9) | 54 (62.1) |  |
| No (*n* = 132) | 29 (22.0) | 103 (78.0) |  |
| Presence of descending colon diverticula |  |  | 0.089 |
| Yes (*n* = 44) | 17 (38.6) | 27 (61.4) |  |
| No (*n* = 175) | 45 (25.7) | 130 (74.3) |  |
| Presence of sigmoid colon diverticula |  |  | 0.002 |
| Yes (*n* = 78) | 32 (41.0) | 46 (59.0) |  |
| No (*n* = 141) | 30 (21.3) | 111 (78.7) |  |
| Charlson comorbidity index. n (%) |  |  | 0.047 |
| ≤ 3 (*n* = 192) | 50 (26.0) | 142 (74.0) |  |
| ≥ 4 (*n* = 27) | 12 (44.4) | 15 (55.6) |  |
| Laboratory findings, median (IQR) |  |  |  |
| Hemoglobin (mg/dL) | 9.9 (8.7–11.8) | 9.8 (8.3–11.6) | 0.296 |
| Platelet count (mm3) | 194 (163–244) | 205 (172–251) | 0.507 |
| PT (INR) | 1.1 (1.0–1.1) | 1.1 (1.0–1.1) | 0.437 |
| eGFR | 86.0 (67.4–99.3) | 80.8 (62.3–93.8) | 0.104 |
| Use of anti-thrombotic agents |  |  | 0.521 |
| Yes (*n* = 109) | 33 (30.3) | 76 (69.7) |  |
| No (*n* = 110) | 29 (26.4) | 81 (73.6) |  |
| Adjustment of anti-thrombotic agents (*n* = 109) |  |  | 0.100 |
| Yes (*n* = 24) | 4 (16.7) | 20 (83.3) |  |
| No (*n* = 85) | 29 (34.1) | 56 (65.9) |  |
| Use of NSAIDs |  |  | 0.266 |
| Yes (*n* = 22) | 4 (18.2) | 18 (81.8) |  |
| No (*n* = 197) | 58 (29.4) | 139 (70.6) |  |
| Hemostatic treatment |  |  | 0.882 |
| Yes (*n* = 62) | 18 (29.0) | 44 (71.0) |  |
| No (*n* = 157) | 44 (28.0) | 113 (72.0) |  |

CDB, colonic diverticular bleeding; DOAC, direct oral anticoagulant; IQR, interquartile range; INR, international normalized ratio; NSAIDs, non-steroidal anti-inflammatory drugs; PT, prothrombin time; rCDB, recurrent CDB; SD, standard deviation.

**Supplementary Table 2.** Comorbidities constituting the Charlson comorbidity index of colonic diverticular bleeding patients

| Variables |  | Total  (*n* = 219) | Patients with rCDB  (*n* = 62) | Patients without rCDB  (*n* = 157) | *p*-value* |
| --- | --- | --- | --- | --- | --- |
| Comorbidities, variable CCI, n (%) | Weight |  |  |  |  |
| Acute myocardial infarction | 1 | 33 (15.1) | 10 (16.1) | 23 (14.6) | 0.78 |
| Congestive heart failure | 1 | 14 (6.4) | 5 (8.1) | 9 (5.7) | 0.53 |
| Peripheral vascular disease | 1 | 11 (4.5) | 1 (1.6) | 9 (5.7) | 0.19 |
| Cerebrovascular accident | 1 | 26 (11.9) | 9 (14.5) | 17 (10.6) | 0.45 |
| Dementia | 1 | 11 (5.0) | 2 (3.2) | 9 (5.7) | 0.44 |
| Chronic pulmonary disease | 1 | 7 (3.2) | 2 (3.2) | 5 (3.2) | 0.99 |
| Connective tissue disease | 1 | 6 (2.7) | 2 (3.2) | 4 (2.5) | 0.78 |
| Ulcer disease | 1 | 12 (5.5) | 2 (3.2) | 10 (6.4) | 0.35 |
| Hepatitis | 1 | 3 (1.4) | 2 (3.2) | 1 (0.6) | 0.14 |
| Diabetes without end-organ damage | 1 | 62 (28.3) | 14 (22.6) | 48 (30.6) | 0.24 |
| Diabetes with end-organ damage | 2 | 15 (6.8) | 7 (11.3) | 8 (5.1) | 0.10 |
| Hemiplegia | 2 | 1 (0.5) | 0 (0) | 1 (0.6) | 0.53 |
| Chronic kidney disease^*^ | 2 | 10 (4.6) | 6 (9.7) | 4 (2.5) | 0.02 |
| Solid tumor without metastasis | 2 | 19 (8.7) | 7 (11.3) | 12 (7.6) | 0.40 |
| Leukemia | 2 | 0 (0) | 0 (0) | 0 (0) | N/A |
| Lymphoma | 2 | 1 (0.5) | 0 (0) | 1(0.6) | 0.53 |
| Liver cirrhosis | 3 | 8 (3.7) | 3 (4.8) | 5 (3.2) | 0.56 |
| Metastatic solid tumor | 6 | 1 (0.5) | 0 (0) | 1 (0.6) | 0.53 |
| Acquired immunodeficiency syndrome | 6 | 0 (0) | 0 (0) | 0 (0) | N/A |

CCI, Charlson comorbidity index; CDB, colonic diverticular bleeding; N/A, not available; rCDB, recurrent CDB.

* Chronic kidney disease was defined as a serum creatinine level > 3.0 mg/dL or if the patient was on dialysis.

**Supplementary Table 3.** Cause of death analysis

|  | Total  (*n* = 33) | Died on first admission  (*n* = 2) | Rebleeding  (*n* = 11) | Non-rebleeding  (*n* = 20) |
| --- | --- | --- | --- | --- |
| Acute myocardial infarction, *n* (%) | 3 (9.1) | 1 (50.0) | 1 (9.1) | 1 (5.0) |
| Acute vascular disease of intestine, *n* (%) | 1 (3.0) | 0 (0) | 0 (0) | 1 (5.0) |
| Atrial fibrillation, *n* (%) | 1 (3.0) | 0 (0) | 0 (0) | 1 (5.0) |
| Asphyxia, *n* (%) | 1 (3.0) | 0 (0) | 0 (0) | 1 (5.0) |
| Brain hemorrhage, *n* (%) | 2 (6.0) | 0 (0) | 0 (0) | 2 (10.0) |
| Cerebral artery dissection, *n* (%) | 1 (3.0) | 0 (0) | 0 (0) | 1 (5.0) |
| Cholangiocarcinoma, *n* (%) | 1 (3.0) | 0 (0) | 0 (0) | 1 (5.0) |
| COPD, *n* (%) | 2 (6.0) | 0 (0) | 1 (9.1) | 1 (5.0) |
| Diverticular bleeding, *n* (%) | 2 (6.0) | 1 (50.0) | 1 (9.1) | 0 |
| Diverticulitis with perforation, *n* (%) | 1 (3.0) | 0 (0) | 0 (0) | 1 (5.0) |
| Diabetes mellitus, *n* (%) | 1 (3.0) | 0 (0) | 1 (9.1) | 0 |
| HCC, *n* (%) | 2 (6.0) | 0 (0) | 1 (9.1) | 1 (5.0) |
| Hypothermia, *n* (%) | 1 (3.0) | 0 (0) | 1 (9.1) | 0 |
| Infection, *n* (%) | 1 (3.0) | 0 (0) | 1 (9.1) | 0 |
| Parkinson’s disease, *n* (%) | 2 (6.0) | 0 (0) | 0 (0) | 2 (10.0) |
| Pneumonia, *n* (%) | 5 (15.2) | 0 (0) | 3 (27.3) | 2 (10.0) |
| Prostate cancer, *n* (%) | 1 (3.0) | 0 (0) | 0 (0) | 1 (5.0) |
| SBP, *n* (%) | 1 (3.0) | 0 (0) | 1 (9.1) | 0 |
| Traffic accident, *n* (%) | 2 (6.0) | 0 (0) | 0 (0) | 2 (10.0) |
| Ventricular fibrillation, *n* (%) | 1 (3.0) | 0 (0) | 0 (0) | 1 (5.0) |
| Unknown origin, *n* (%) | 1 (3.0) | 0 (0) | 0 (0) | 1 (5.0) |

COPD, chronic obstructive pulmonary disease; HCC, hepatocellular carcinoma; SBP, spontaneous bacterial peritonitis.
